# The Critical Role of Chemokine (C–C Motif) Receptor 2-Positive Monocytes in Autoimmune Cholangitis

**DOI:** 10.3389/fimmu.2018.01852

**Published:** 2018-08-15

**Authors:** Debby Reuveni, Yael Gore, Patrick S. C. Leung, Yael Lichter, Itay Moshkovits, Ayelet Kaminitz, Eli Brazowski, Eric Lefebvre, Pamela Vig, Chen Varol, Zamir Halpern, Oren Shibolet, Merrill Eric Gershwin, Ehud Zigmond

**Affiliations:** ^1^The Research Center for Digestive Tract and Liver Diseases, Tel Aviv Sourasky Medical Center, Tel Aviv, Israel; ^2^Sackler Faculty of Medicine, Tel Aviv University, Tel Aviv, Israel; ^3^Division of Rheumatology, Allergy and Clinical Immunology, University of California, Davis, Davis, CA, United States; ^4^Department of Pathology, Tel Aviv Sourasky Medical Center, Tel Aviv, Israel; ^5^Allergan Plc, South San Francisco, CA, United States

**Keywords:** monocytes, macrophages, chemokine, therapy, primary biliary cholangitis

## Abstract

The therapy of primary biliary cholangitis (PBC) has lagged behind other autoimmune diseases despite significant improvements in our understanding of both immunological and molecular events that lead to loss of tolerance to the E2 component of pyruvate dehydrogenase, the immunodominant autoepitope of PBC. It is well known that Ly6C^hi^ monocytes are innate immune cells infiltrating inflammatory sites that are dependent on the expression of C–C motif chemokine receptor 2 (CCR2) for emigration from bone marrow. Importantly, humans with PBC have a circulating monocyte pro-inflammatory phenotype with macrophage accumulation in portal tracts. We have taken advantage of an inducible chemical xenobiotic model of PBC and recapitulated the massive infiltration of monocytes to portal areas. To determine the clinical significance, we immunized both CCR2-deficient mice and controls with 2OA-BSA and noted that CCR2 deficiency is protective for the development of autoimmune cholangitis. Importantly, because of the therapeutic potential, we focused on inhibiting monocyte infiltration through the use of cenicriviroc (CVC), a dual chemokine receptor CCR2/CCR5 antagonist shown to be safe in human trials. Importantly, treatment with CVC resulted in amelioration of all aspects of disease severity including serum total bile acids, histological severity score, and fibrosis stage. In conclusion, our results indicate a major role for Ly6C^hi^ monocytes and for CCR2 in PBC pathogenesis and suggest that inhibition of this axis by CVC should be explored in humans through the use of clinical trials.

## Introduction

Primary biliary cholangitis (PBC) is a chronic autoimmune cholestatic liver disease with a female predominance characterized by nonsuppurative lymphocytic cholangitis causing immune-mediated destruction of intrahepatic bile ducts leading to progressive cholestasis, biliary fibrosis, and eventually cirrhosis (1). The etiology of PBC is unknown, but it is believed that genetic susceptibility and environmental factors are dually involved. Hence, environmental, infectious, genetic, epigenetic, metabolic, and immunological factors may account for the breakdown of immunological tolerance, and a number of xenobiotic and infectious agents have been proposed to induce the disease in genetically predisposed individuals (2). Anti-mitochondrial antibodies (AMAs), which specifically target lipoyl domain of the E2 subunit of the mitochondrial enzyme pyruvate dehydrogenase (PDC-E2), as well as PDC-specific T cells are found in 95% of the patients (3, 4). Mapping of the epitopes recognized by the cellular and humoral immune response against PDC-E2 led to the observation that these sites are highly conserved molecular sequences flanking lipoic acid-binding sites (5). Of note, anti-PDC-E2 antibodies from PBC patients recognize xenobiotic modified PDC-E2 peptides that mimic lipoic acid and results in higher titer reactivity than the native autoantigen. Importantly, detailed quantitative structure–activity relationship analysis identified 2-octynoic acid (2OA) as an even better xenobiotic candidate for antigenic modification of the PDC-E2 peptide (5–7). In this respect, we have established a murine model for PBC based on immunization of mice with 2OA conjugated to bovine serum albumin (2OA-BSA) resulting in the appearance of anti-PDC-E2 antibodies and histological lesions typical of autoimmune cholangitis (8).

Accumulative data suggest involvement of the innate immune system in PBC pathogenesis (9). The comprehension of liver macrophages has been revolutionized by the delineation of heterogeneous subsets of these cells exhibiting functional plasticity. Kupffer cells (KCs) are a self-sustaining, liver-resident population of macrophages and can be distinguished from the recruited monocyte-derived macrophages (MoMFs), which arise from circulating Ly6C^hi^ monocytes that rapidly accumulate in the diseased liver (10). With respect to the latter, monocytes are members of the mononuclear phagocyte system and constitutes a critical effector component of the innate immune arm. Classical Ly6C^hi^ monocytes originate from a common myeloid progenitor cell in the bone marrow, emigrate from the bone marrow in a C–C motif chemokine receptor 2 (CCR2)-dependent manner, and released into the bloodstream as non-differentiated cells that circulate in the blood for 1–3 days (11–14). Following their recruitment to tissues, monocytes undergo differentiation into tissue macrophages or myeloid dendritic cells (15). Following arrival to their destination, monocyte or cells differentiated from monocytes conduct versatile functions including initiation and progression of inflammation, homeostasis maintenance, host defense, and tissue remodeling and repair, depending on specific environmental signals (16–19). Several studies have suggested altered monocyte/macrophage function in PBC and accumulation of these cells in the livers of PBC patients (20, 21). Mononuclear cells expressing the low-density lipoprotein binding glycoprotein CD68 were detected in the biliary epithelial layer and periductal tissue of PBC patients, whereas in patients with viral hepatitis, these cells were scattered, and in normal livers were rarely seen around bile ducts (21). These studies suggest involvement of monocyte-derived cells in PBC pathogenesis; however, solid data evaluating the importance and function of these cells in the *in vivo* context were missing so far.

Here, we demonstrate, by using a xenobiotic murine model of PBC, that infiltrating Ly6C^hi^ monocytes are massively recruited into the portal zone of livers in a CCR2-dependent fashion. CCR2-deficient mice subjected to this model exhibit milder disease phenotype. Consequently, pharmacological inhibition of monocyte infiltration by blocking CCR2/CCR5 with Cenicriviroc attenuated disease severity as assessed by serum bile acids, histological severity scoring, and liver fibrosis quantification. Our results indicate a major role for monocyte-derived cells in PBC pathogenesis and proved that targeted inhibition of these cells significantly ameliorated both liver inflammation and fibrosis. These results reinforce further investigation of this therapeutic option in clinical trials.

## Materials and Methods

### Mice

Female C57BL/6 WT mice were purchased from ENVIGO. *Cx3cr1^gfp/+^, Ccr2^−/−^Cx3cr1^gfp/+^* mice were kindly provided by Prof. Steffen Jung, the Weizmann Institute of Science and maintained in the animal facility of the Tel-Aviv Sourasky Medical Center. Mice had unrestricted access to food and water, were housed in temperature and humidity-controlled rooms, and were kept on a 12-h light/dark cycle. Use of animals was in accordance with the National Institutes of Health policy on the care and use of laboratory animals and was approved by the Tel-Aviv Sourasky Medical Center Animal Use and Care Committee.

### Preparation of Immunogen

2-octynoic acid was purchased from Sigma-Aldrich (St. Louis, MO, USA) and was conjugated with BSA (EMD Chemicals, Gibbstown, NJ, USA), as described previously (8). Briefly, 2OA was dissolved in dry dimethyl ether. *N*-hydroxysuccinimide (NHS) was then added, and the solution was cooled to 0°C and stirred for 20 min. Dicyclohexylcarbodiimide was then added, and the mixture was allowed to warm to ambient temperature overnight. The solution was filtered, concentrated by roto-evaporation under reduced pressure, re-dissolved with ethyl ether, washed with water, NaHCO_3_ (1 M), brine, dried under magnesium sulfate, filtered, and concentrated. The product was then purified using flash chromatography (30% ethyl acetate/hexane). NHS-activated 2OA was dissolved in dimethyl sulfoxide and then coupled to the lysine residues of BSA. The solution was allowed to react for 3 h followed by dialysis [phosphate-buffered saline (PBS)].

### Immunization and CVC Treatment

Female mice at 8 weeks of age were immunized with 2OA-BSA at 1 mg/ml per animal intraperitoneally (i.p.) in the presence of complete Freund’s adjuvant (CFA) (Sigma-Aldrich) containing 10 mg/ml of *Mycobacterium tuberculosis* strain H37Ra. In addition, pertusis toxin (Sigma-Aldrich, 100 ng/animal) was delivered i.p. at the day of immunization and 2 days after. All animals were given boost immunizations 2 weeks following the initial immunization with 2OA-BSA in incomplete Freund’s adjuvant (Sigma-Aldrich). Animals were sacrificed 8–12 weeks after initial immunization.

For testing the efficacy of CVC, immunized mice received daily i.p. injections of CVC (20 mg/kg) (Allergen plc) or vehicle only as control, starting the day after the boosting until the end of the experiment (8 weeks from initial immunization).

### Detection of Serum AMAs

Serum AMAs were detected using enzyme-linked immunosorbent assay (ELISA) utilizing recombinant human pyruvate dehydrogenase complex-E2 (PDC-E2) to coat individual wells of a 96-well microtiter plate (8). Samples were tested at a dilution of 1:1,000. Immunoreactivity was determined by measuring the optical density at 450 nm after incubation with 100 µl of 3,3′,5,5′-tetramethylbenzidine (Merck, Kenilworth, NJ, USA) for 30 min followed by the addition of 50 µl of 18N H_2_SO_4_ stopping solution.

### Histopathology

Livers from euthanized mice were fixed in formaldehyde 4% buffered (pH 7.2), embedded in paraffin, cut into 4-µm sections, deparaffinized, and stained with hematoxylin and eosin. Disease severity was evaluated by experience pathologist (Eli Brazowski) who was blinded to treatment allocation. Scoring was done considering four parameters (portal infiltrate, bile duct damage and loss, granulomas formation, and lobular inflammation) ranging between 0 and 2 (normal, moderate, or severe, respectively).

### Fibrosis Evaluation

Picro-Sirius red staining was performed using a commercially available kit (ab150681, Abcam, Cambridge, UK). The staining procedure was carried out according to the manufacturer’s protocol. Briefly, deparaffinized sections were stained with Picro-Sirius red solution for 1 h. Slides were then rinsed twice in acetic acid solution, followed by dehydration with absolute alcohol and mounting in synthetic resin.

Tissue collagen accumulation was quantitatively assessed by a hydroxyproline accumulation assay (Sigma-Aldrich; MAK008) according to the manufacturer’s instructions.

### Isolation of Hepatic Non-Parenchymal Cells for Flow Cytometry

Isolation of hepatic non-parenchymal cells was performed as previously described (22). In brief, mice were anesthetized; the livers were perfused with cold PBS and excised. Livers were cut into small fragments, incubated (37°C, 250 rpm for 45 min) with 5 ml digestion buffer [5% FBS, 0.5 mg/ml collagenase IV (Sigma-Aldrich, Rehovot, Israel, C5138-500MG), 0.1 mg/ml deoxyribonuclease I from bovine pancreas (Sigma-Aldrich, USA) in PBS], and filtered through 200-µm wire mesh. This was followed by three cycles of washings with PBS at 400 rpm, 4°C, 5 min, harvesting the supernatant, and discarding the parenchymal cell pellet. The supernatant was centrifuged at 1,500 rpm, 4°C, 5 min. The erythrocytes in the cell pellet were lysed by 2 min incubation with ACK Lysing buffer (0.15 M NH_4_Cl, 0.01 M KHCO_3_) and washed with PBS.

### Flow Cytometry Analysis

Cell preparations were incubated with monoclonal antibody 2·4G2 for FcR blocking (BioLegend, San Diego, CA, USA) and then exposed at 4°C to a combination of the following antibodies: anti-mouse CD45 (clone 30-F11), anti-mouse/human CD11b (clone M1/70), anti-mouse Ly6C (clone HK1.4), anti-mouse MHCII (clone M5/114.15.2), anti-mouse CD64 (clone X54-5/7.1), anti-mouse CD3ε (clone 145-2c11), anti-mouse CD8a (clone 53-6.7), anti-mouse CD4 (clone GK1.5), and anti-mouse TCRβ (clone 457-597) all were purchased from BioLegend, San Diego, CA, USA. Anti-mouse F4/80 (clone A3-1) was purchased from BIORAD. Cells were analyzed with BD FACSCanto™ II (BD Bioscience). Flow cytometry analysis was performed using Flow Jo Software (Tree Star, Ashland, OR, USA).

### Immunofluorescence of Frozen Tissue Sections

Cx3cr1-GFP immunofluorescent was performed on frozen liver sections of 13 µm. Slides were incubated in cold acetone for 7 min and dried at room temperature. Samples were closed with Fluorescent Mounting Medium with or without 4′,6′-diamidino-2-phenylindole (DAPI, GBI Labs). Images were taken with ZEISS Confocal Microscope (MicroImaging GmbH, ZEISS, Germany). Processing was performed with ZEN 2010 Software.

### Quantitative Real-Time PCR

RNA from livers was isolated with an RNeasy Micro kit (QIAGEN) and reverse transcribed with a High Capacity cDNA Reversed transcription kit (Applied Biosystems) according to the manufacturer’s instructions. PCRs were performed with the SYBER green PCR Master Mix (Applied Biosystems). Quantification was done with Step One Software (V2.2). The genes of interest: collagen 1 (Coll1), transforming growth factor beta (Tgfβ), tissue inhibitor of metalloproteinase 1 (TIMP-1), alpha smooth muscle actin (αSMA), Ifnγ, tumor necrosis factor alpha (Tnfα), Ccl2, Cx3cl1, and Il1β were compared with ribosomal protein large P0 housekeeping gene. Primer sequences (forward and reverse, respectively) were:

RPLPO, 5′-TCCAGCAGGTGTTTGACAAC-3′ and 5′-CCATCTGCAGACACACACT-3′;
Coll-I, 5′-GAGAGCATGACCGATGGATT-3′ and 5′-CCTTCTTGAGGTTGCCAGTC-3′;
TGFβ, 5′-ATTCAGCGCTCACTGCTCTT-3′ and 5′-GTTGGTATCCAGGGCTCTCC-3′;
TIMP-1, 5′-TCCCCAGAAATCAACGAGAC-3′ and 5′-CTGGGACTTGTGGGCATATC-3′;
αSMA, 5′-TGATCACCATTGGAAACGAA-3′ and 5′-CCCCTGACAGGACGTTGTTA-3′;
TNFα, 5′-CGAGTGACAAGCCTGTAGCC-3′ and 5′-CCTTGTCCCTTGAAGAGAACC-3′;
IFNγ, 5′-GCGTCATTGAATCACACCTG-3′ and 5′-TGAGCTCATTGAATGCTTGG-3′;
CCL-2, 5′-AGGTCCCTGTCATGCTTCTG-3′ and 5′-GCTGCTGGTGATCCTCTTGT-3′;
CX3CL1, 5′-GCTGTGACGCGATGCACTTTC-3′ and 5′-CACTGGCACCAGGACGTATG-3′;
IL1-β, 5′-GACCTTCCAGGATGAGGACA-3′ and 5′-AGCTCATATGGGTCCGACAG-3′.

### Cell Line

Human acute monocytic leukemia cell line THP-1 (ATCC TIB 202) was maintained in RPMI-1640 medium supplemented with 10% heat-inactivated fetal bovine serum, 2 mM l-glutamine, 100 U/ml penicillin, and 100 mg/ml streptomycin at 37°C in a humidified incubator with 5% CO_2_.

### TNF-α Levels Analysis by ELISA

THP-1 cells suspended in RPMI medium, as described above, were placed in 24-well culture plates at a concentration of 1 × 10^6^ cells/ml and incubated for 24 h with various concentrations of CVC (0.1–10 µM) prior to LPS stimulation (10–100 ng/ml) for 3 h. Cell-free supernatants were recovered, and the concentrations of TNF-α were measured using a commercially available sandwich ELISA kit according to the manufacturer’s instructions (R&D Systems Inc.).

### Statistical Analysis

The results are presented as mean ± SEM. Statistical significance was assessed using a two-tailed Student’s *t*-test; *p* values <0.05 were considered as significant.

## Results

### Characterization of Hepatic Macrophages in Steady State and Experimental Chronic Autoimmune Cholangitis

**Figure 1:**
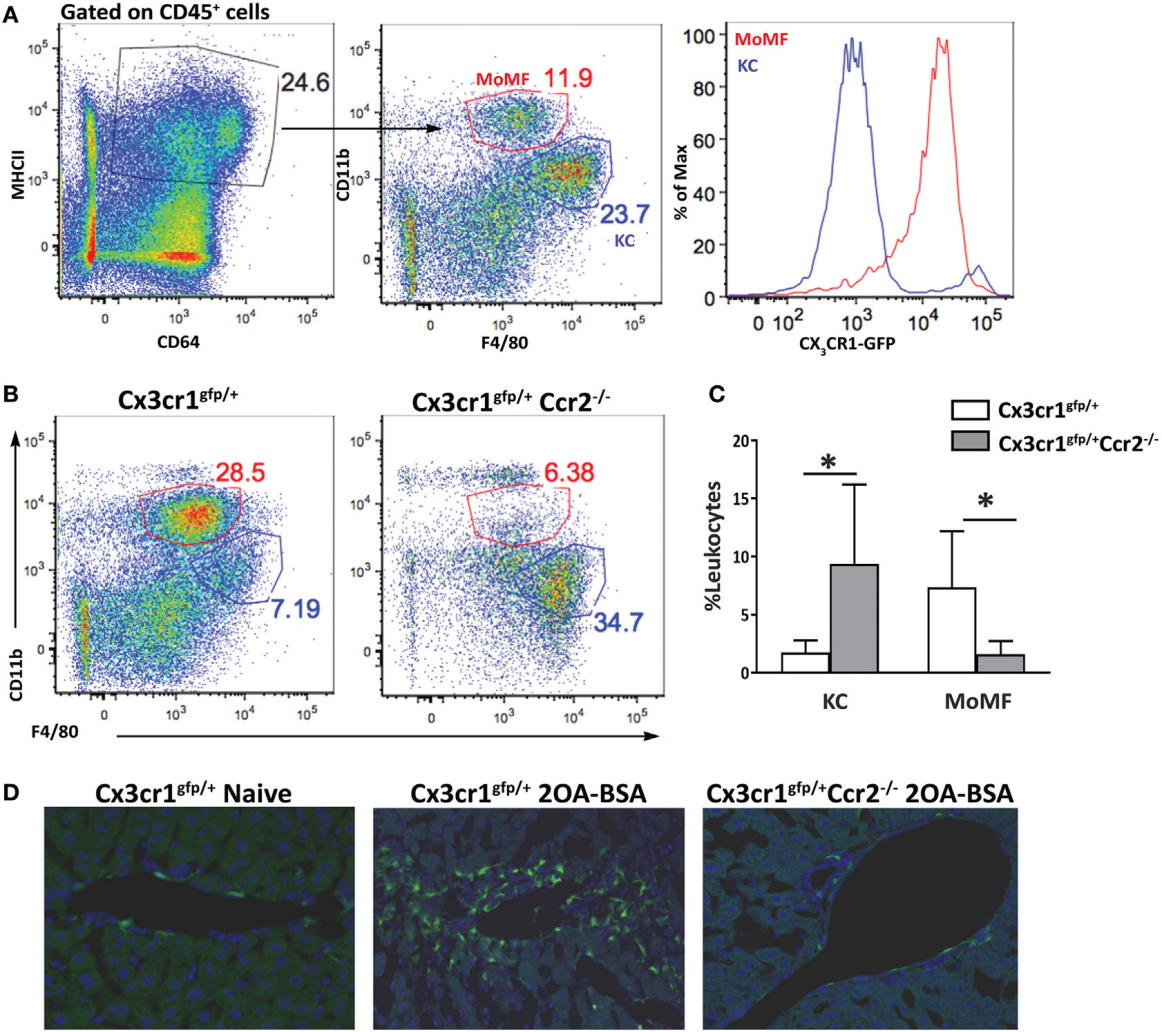
Monocyte-derived cells accumulate around bile ducts in the murine 2-octynoic acid conjugated to bovine serum albumin (2OA-BSA) autoimmune cholangitis model. **(A)** Flow cytometry analysis of non-parenchymal liver cells of Cx3cr1^gfp/+^ mice at steady state. **(B)** Flow cytometry analysis of non-parenchymal liver cells of Cx3cr1^gfp/+^ and Cx3cr1^gfp/+^Ccr2*^−/−^* mice, 8 weeks following immunization with 2OA-BSA. **(C)** Graphical summary of flow cytometry analysis of non-parenchymal liver cells of indicated mice, 8 weeks post immunization with 2OA-BSA. **(D)** Representative fluorescent microscopy images of livers from Cx3cr1^gfp/+^ and Cx3cr1^gfp/+^Ccr2*^−/−^* mice 8 weeks post immunization with 2OA-BSA and naïve Cx3cr1^gfp/+^ mice (Green—CX3CR1-GFP, Blue—DAPI). Data are presented as mean ± SEM (*n* ≥ 10). *p < 0.05 (unpaired Student’s *t*-test).

The liver macrophage compartment harbors a main self-maintaining population of resident macrophages named KCs and an infiltrating population of MoMFs that accumulate to be the dominant liver macrophage population under various pathological settings. Others and we have demonstrated that infiltrating MoMFs can be distinguished from resident KCs by their expression of the fractalkine chemokine receptor (CX_3_CR1) (22, 23). To identify and characterize macrophages populations in the liver under the setting of autoimmune cholangitis, we took advantage of *Cx3cr1^gfp/+^* reporter mice (24). *Cx3cr1^gfp/+^* and *Cx3cr1^gfp/+^Ccr2^−/−^* mice were subjected to the 2OA-BSA PBC model and 8 weeks following the initial immunization liver macrophages subsets were evaluated. As previously reported, flows cytometry analysis of non-parenchymal liver cells in steady-state livers revealed two main types of CD64^+^MHC II^+^ macrophages: CD11b^int^F4/80^hi^CX_3_CR1^neg^ KCs and the CD11b^hi^F4/80^int^CX3CR1^hi^ MoMFs (Figure 1A). In vast contrast, livers from the immunized mice exhibited profound accumulation of MoMFs and reduction in the occurrence of KCs. As predicted, in CCR2-deficient mice, monocyte-derived cells did not infiltrate the chronically inflamed liver (Figures 1B,C). Visualization of liver section derived from immunized mice by fluorescent microscopy, revealed accumulation of CX3CR1-GFP-positive cells around the portal triads of 2OA-BSA immunized *Cx3cr1^gfp/+^* mice, whereas in *Cx3cr1^gfp/+^Ccr2^−/−^* immunized mice and *Cx3cr1^gfp/+^* naive mice very few CX3CR1-GFP-positive cells were detected in the portal area (Figure 1D).

### CCR2-Deficient Mice Display Attenuated Disease Severity in the 2OA-BSA Autoimmune Cholangitis Model

**Figure 2:**
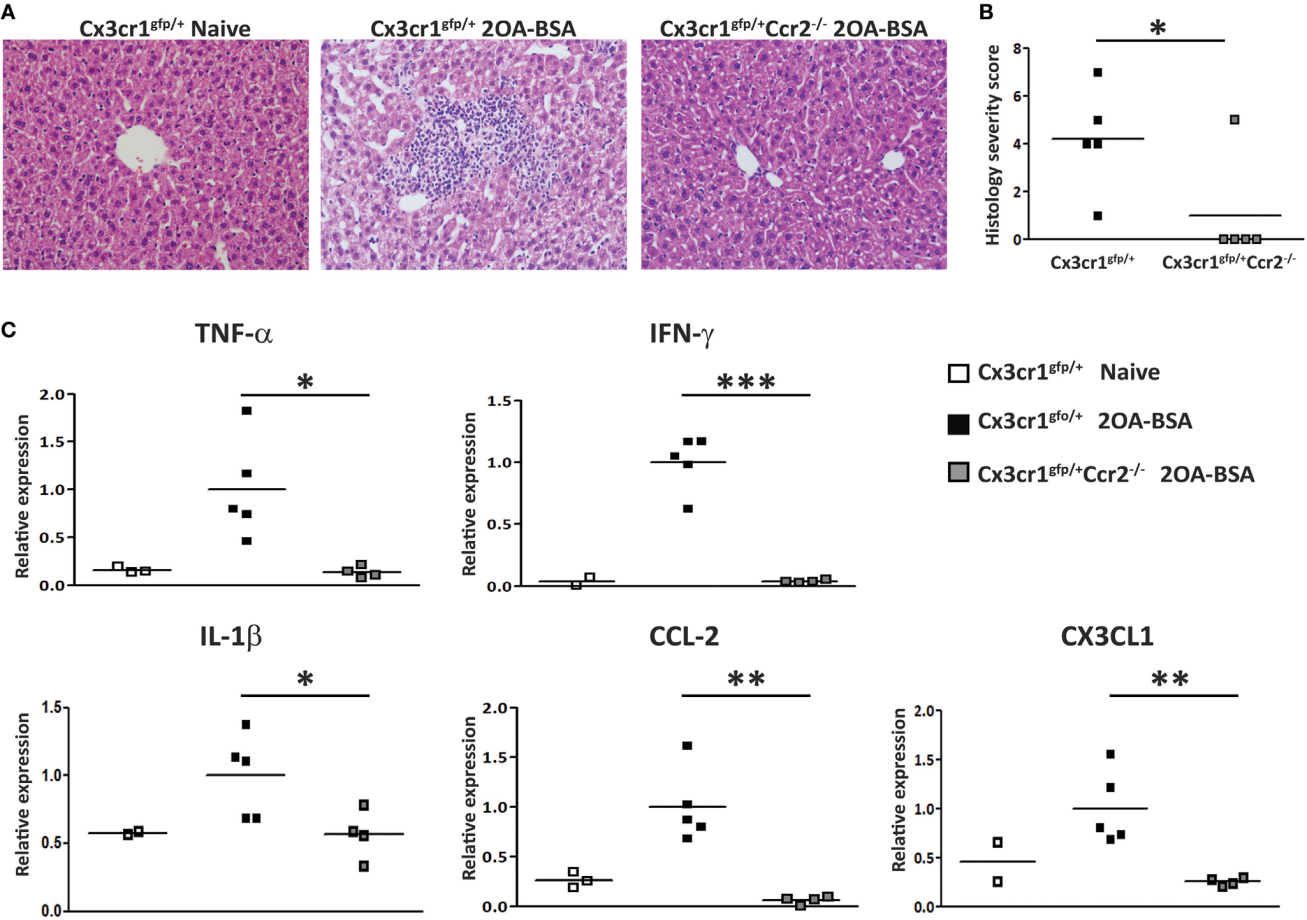
C-C motif chemokine receptor 2-deficient mice display attenuated disease severity in the 2-octynoic acid conjugated to bovine serum albumin (2OA-BSA) autoimmune cholangitis model. **(A)** Representative liver histology (hematoxylin and eosin staining) of indicated mice, original magnification 40×. **(B)** Graphical summary of histological disease severity score. **(C)** Graphical summary of hepatic cytokines expression levels of indicated mice. All data are presented as mean, *n* ≥ 5 per experimental group. Results are representative of three or more independent experiments. *p < 0.05 and **p < 0.01 (unpaired Student’s *t*-test).

To evaluate the impact of MoMF deficiency on autoimmune cholangitis, liver sections of *Cx3cr1^gfp/+^* reporter mice and *Cx3cr1^gfp/+^Ccr2^−/−^* mice immunized with 2OA-BSA were examined. *Cx3cr1^gfp/+^* mice demonstrated periportal infiltration of lymphocytes and mononuclear cells and bile ducts destruction, whereas, in *Cx3cr1^gfp/+^Ccr2^−/−^* mice, no abnormalities were observed (Figure 2A). Histological disease severity scoring revealed a significant attenuation in disease severity in CCR2-deficient mice (Figure 2B). Serum AMA titers were comparable in *Cx3cr1^gfp/+^* reporter mice and *Cx3cr1^gfp/+^Ccr2^−/−^* mice immunized subjected to the 2OA-BSA model and found to be significantly higher then in the serum of naïve mice (Figure S1A in Supplementary Material).

To further investigate the effect of MoMF deficiency on liver inflammation, expression of pro-inflammatory cytokines and chemokines was examined in liver tissue using quantitative real time PCR. The levels of *Tnf*α, *Ifnγ, Il1β, Ccl2*, and *Cx3cl1* were significantly increased in *Cx3cr1^gfp/+^* mice but not in *Cx3cr1^gfp/+^Ccr2^−/−^* mice subjected to the 2OA-BSA model, indicating that MoMFs profoundly contribute to the development of pro-inflammatory response in the immunized livers (Figure 2C).

### CCR2-Deficient Mice Are Protected From the Development of Liver Fibrosis in the 2OA-BSA Autoimmune Cholangitis Model

**Figure 3:**
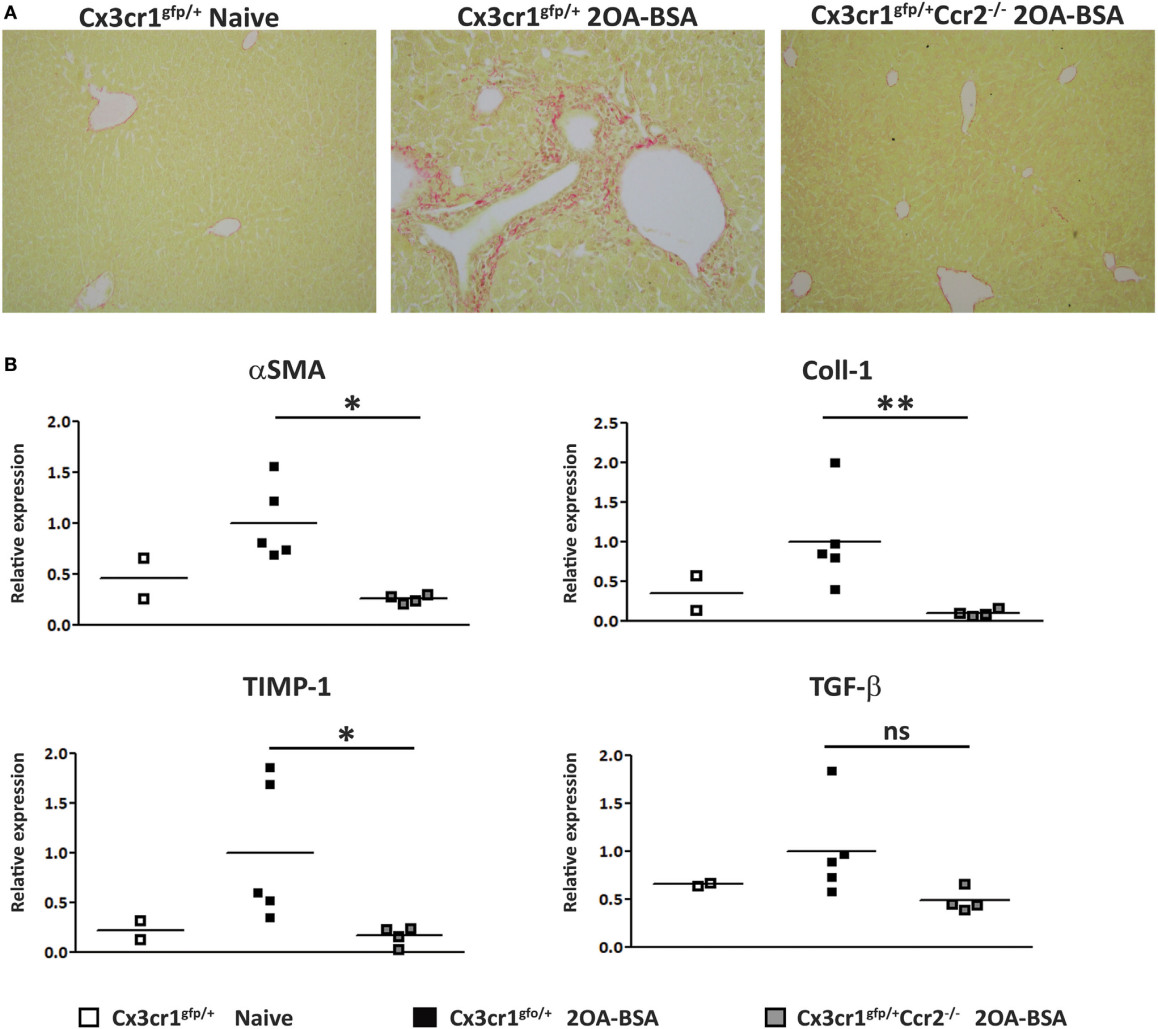
C-C motif chemokine receptor 2-deficient mice are protected from the development of fibrosis in the 2-octynoic acid conjugated to bovine serum albumin (2OA-BSA) autoimmune cholangitis model. **(A)** Representative liver sections from indicated mice—Picro-Sirius red staining. **(B)** Graphical summary of hepatic expression levels of fibrosis-associated genes of indicated mice. All data are presented as mean, *n* ≥ 5 per experimental group. Results are representative of three or more independent experiments. *p < 0.05 and **p < 0.01 (unpaired Student’s *t*-test).

To assess the extent of liver fibrosis, Sirius red staining for detection of collagen distribution was performed at 8 weeks following immunization with 2OA-BSA. *Cx3cr1^gfp/+^* mice exhibited periportal collagen deposition while *Cx3cr1^gfp/+^Ccr2^−/−^* or *Cx3cr1^gfp/+^* control mice displayed normal collagen distribution similar to the non-immunized *Cx3cr1^gfp/+^* control mice (Figure 3A). This finding was supported by a lower pro-fibrotic hepatic gene expression signature in *Cx3cr1^gfp/+^Ccr2^−/−^* mice including significant reduced expression levels of genes encoding for αSMA, Coll1, and TIMP-1 in CCR2-deficient mice (Figure 3B). There was also clear reduction trend in the expression levels of the gene encoding for TGFβ, previously pointed as a key pro-fibrotic factor provided by infiltrating Ly6C^hi^ monocytes (25).

### Pharmacological Inhibition of CCR2/CCR5 Blocks Monocytes Infiltration and Attenuates Experimental Autoimmune Cholangitis

In light of our findings regarding the role of CCR2 in the development of autoimmune cholangitis, we aimed at assessing the efficacy of the dual CCR2/CCR5 inhibitor Cenicriviroc (CVC) as a potential treatment for this pathology (26). *WT—C57BL/6* and *Cx3cr1^gfp/+^* reporter mice were subjected to the 2OA-BSA model. CVC treatment was initiated 1 day after 2OA-BSA boosting (day 15) by daily intra-peritoneal injections (2 mg/kg), for the remaining 6 weeks. Control mice received vehicle only.

**Figure 4:**
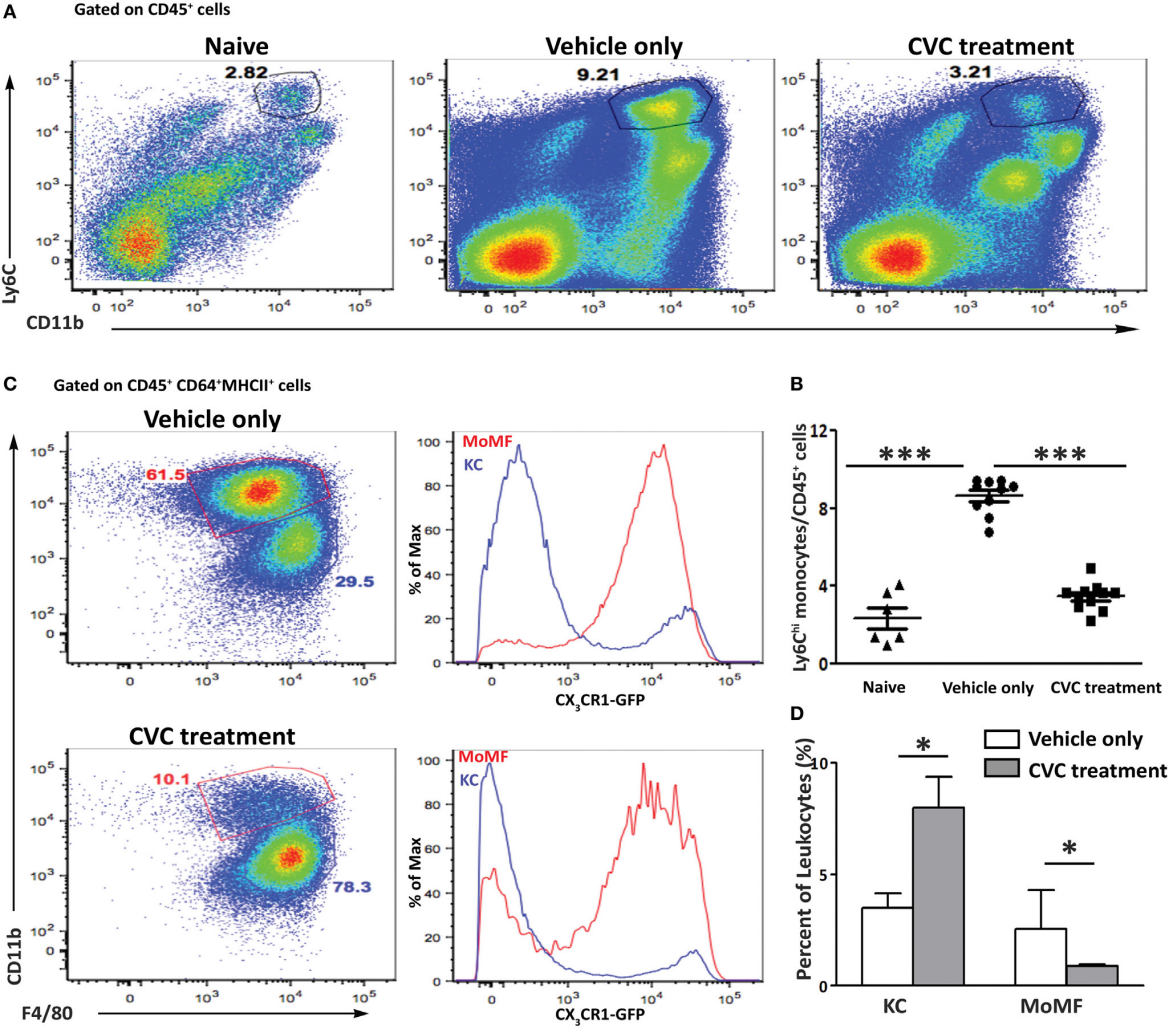
Reduced hepatic infiltration of monocyte-derived cell in CVC-treated mice subjected to the 2-octynoic acid conjugated to bovine serum albumin (2OA-BSA) autoimmune cholangitis model. **(A)** Representative flow cytometry analysis of non-parenchymal liver cells 8 weeks following immunization with 2OA-BSA, in indicated groups of mice. **(B)** Graphical summary of flow cytometry analysis shown in panel **(A)**. **(C)** Flow cytometry analysis of non-parenchymal liver cells of *Cx3cr1^gfp/+^* reporter mice 8 weeks following 2OA-BSA immunization treated with CVC or vehicle only. **(D)** Graphical summary of flow cytometry analysis of non-parenchymal liver cells of *C57BL/6* mice 8 weeks post immunization with 2OA-BSA treated with CVC or vehicle only. Results are mean ± SEM (*n* ≥ 10) for each group. Results are representative of two independent experiments. *p < 0.05 and ***p < 0.001 (unpaired Student’s *t*-test).

Flow cytometry analysis of non-parenchymal liver cells demonstrated a significant reduction in the occurrence of infiltrating Ly6C^hi^ monocytes in the CVC-treated mice (Figures 4A,B). This was further manifested by the reduced fraction of the MoMFs descendants of infiltrating Ly6C^hi^ monocytes. CVC treatment was specific to the Ly6C^hi^ monocyte-derived liver macrophages as there was increased representation of resident KCs in livers from CVC-treated mice (Figures 4C,D).

**Figure 5:**
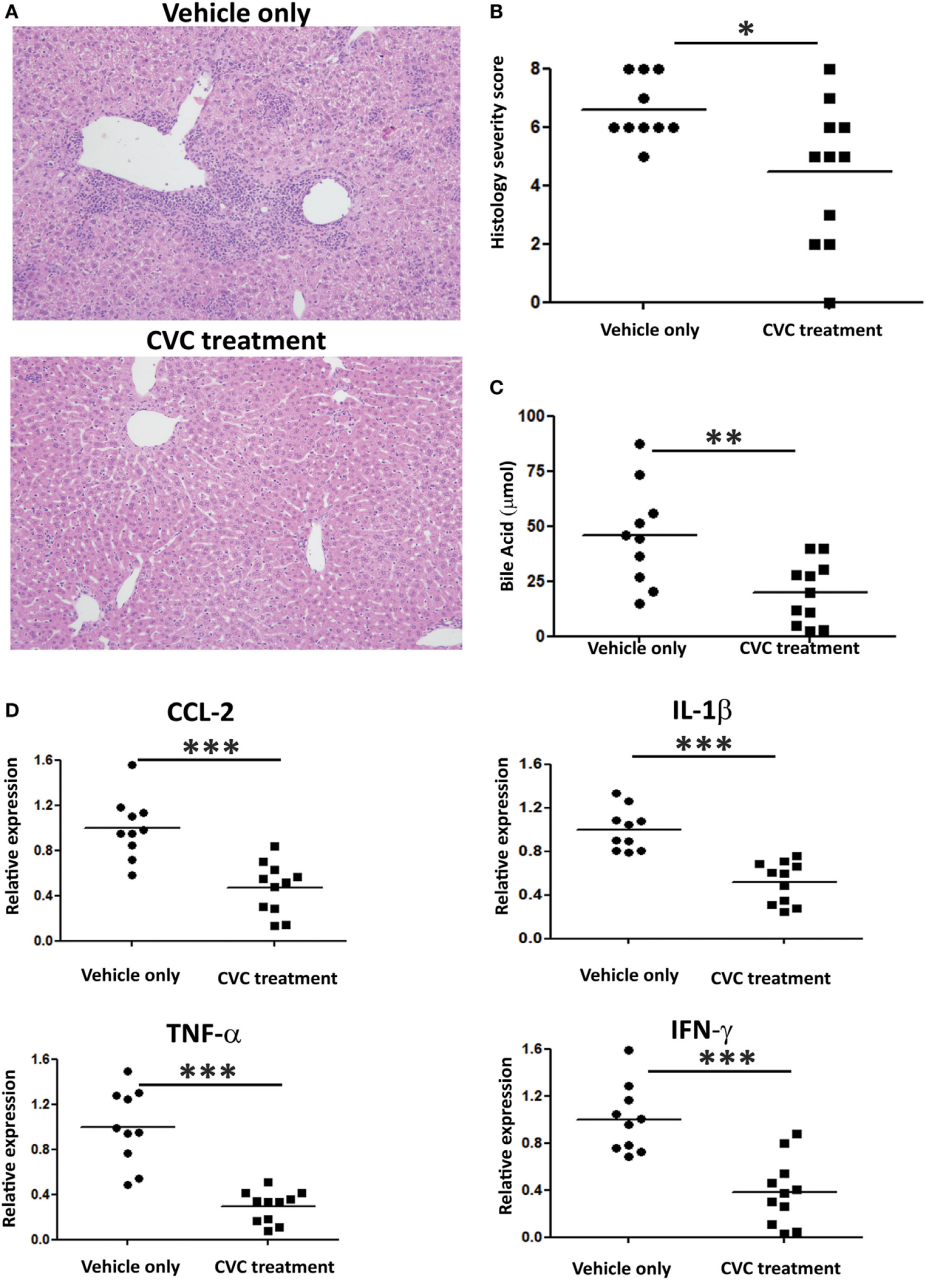
CVC treatment ameliorates disease severity in the 2-octynoic acid conjugated to bovine serum albumin autoimmune cholangitis model. **(A)** Representative liver histology (hematoxylin and eosin staining) of indicated groups of mice, original magnification 40×. **(B)** Graphical summary of histological disease severity score. **(C)** Biochemical profile of total bile acid in indicated mice groups. **(D)** Graphical summary of hepatic cytokines expression levels of indicated mice groups. All data are presented as mean (*n* ≥ 10). Results are representative of two independent experiments. *p < 0.05, **p < 0.01, and ***p < 0.001 (unpaired Student’s *t*-test).

Next, we evaluated the effect of CVC treatment on parameters of disease severity. Histologic evaluation of liver sections from CVC-treated mice revealed normal liver tissue with almost no infiltration of leukocytes, whereas mice administered with vehicle only exhibited periportal infiltration and accumulation of immune cells around the bile ducts (Figure 5A). Serum AMA titers were comparable in CVC and vehicle-treated groups subjected to the 2OA-BSA model and found to be significantly higher then in the serum of naïve mice, indicating that this intervention did not interfere with AMA production (Figure S1B in Supplementary Material). Blinded histological severity score assessment revealed significant improvement in CVC-treated mice (Figure 5B). In addition, serum level of total bile acid, a biochemical marker of PBC, was markedly decreased in mice treated with CVC (Figure 5C). Assessment of hepatic pro-inflammatory cytokines and chemokines milieu revealed lower gene expression levels of *Tnfα, Ifnγ, Ccl2*, and *Il1β* in CVC-treated mice (Figure 5D).

### Cenicriviroc Ameloriates Liver Fibrosis in the 2OA-BSA Autoimmune Cholangitis Model

**Figure 6:**
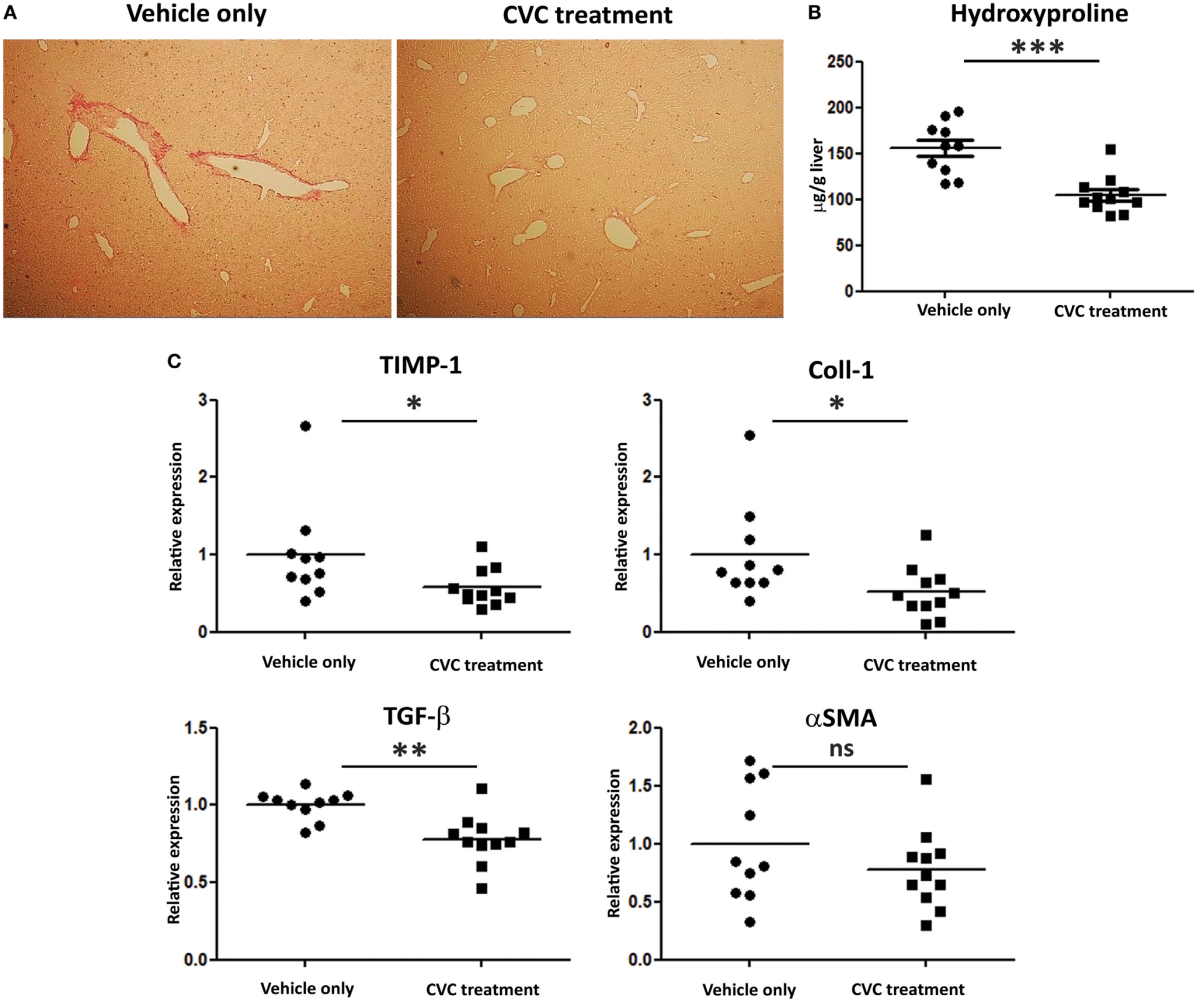
CVC treatment prevents the development of hepatic fibrosis in the 2-octynoic acid conjugated to bovine serum albumin autoimmune cholangitis model. **(A)** Representative liver sections from indicated mice—Picro-Sirius red staining. **(B)** Graphical summary of hepatic hydroxyproline content quantification in indicated mice groups. **(C)** Graphical summary of hepatic expression levels of fibrosis-associated genes of indicated mice groups. Results are mean (*n* ≥ 10) for each group. Results are representative of two independent experiments. *p < 0.05, **p < 0.01, and ***p < 0.001 (unpaired Student’s *t*-test).

Evaluation of fibrosis severity was performed by Sirius red staining, hepatic hydroxyproline quantification, and expression levels analysis of pro-fibrotic genes in the liver (Figures 6A–C, respectively). While portal fibrosis was clearly evident in the livers of vehicle-treated group, livers from CVC-treated mice displayed normal collagen deposition (Figure 6A). In addition, hydroxyproline levels were significantly higher in the vehicle-treated group versus CVC-treated group, indicating higher collagen content in vehicle-treated mice (Figure 6B). Furthermore, real time-PCR analyses for pro-fibrotic genes in liver samples revealed significantly higher expression levels of genes encoding for TIMP-1, Coll1, and TGFβ in vehicle-treated versus CVC-treated mice (Figure 6C). Collectively, these results highlight a therapeutic potency for CVC in the amelioration of hepatic fibrosis in experimental PBC.

## Discussion

Here, we explored the role of CCR2-positive monocytes in the development of experimental PBC. We showed that these cells massively infiltrate the liver to become the dominant hepatic macrophage population in the setting of chronic cholangitis. Inhibition of the recruitment of these cells either by genetic deficiency in CCR2-deficient mice or by pharmacological inhibition of this pathway resulted in amelioration of all aspects of the pathology including portal inflammation, biochemical markers, and fibrosis.

In the setting of acute and chronic inflammation, monocyte infiltrate has the potential to aggravate the pathology by various mechanism including antigen presentation and secretion of various mediators including pro-inflammatory cytokine and factors supporting angiogenesis and fibrosis (19, 27, 28). The mechanism Ly6C^hi^ monocytes and cells derived from these cells contribute to autoimmune cholangitis is probably multifactorial and has not been elucidated in this study. Future studies utilizing *in vivo* tools that target-specific functions of these cells are warranted and might discover new targets to treat this pathology. Of note, other murine models of hepatic fibrosis uncovered opposing functions of hepatic macrophages during progression and regression of fibrosis. Therefore, depletion of macrophages could not only prevent fibrosis development but also delayed repair processes after cessation of the injury (29). It has been suggested that fibrogenic macrophages can switch their phenotype toward restorative macrophages, which are characterized by high expression of anti-inflammatory mediators and matrix-degrading metalloproteinases (30). Thus, the overall effect of long-term inhibition of monocyte recruitment to the liver in PBC will have to be evaluated in appropriate clinical trial.

Cenicriviroc (CVC) is an orally available, dual inhibitor of the chemokine receptors CCR2 and CCR5 (26). CVC treatment has been recently shown to ameliorate disease severity in experimental models of liver disorders including nonalcoholic steatohepatitis and paracetamol induced liver injury (31, 32). A phase 2b trial of CVC in patients with non-alcoholic steatohepatitis (NASH) showed improved fibrosis with safety and tolerability comparable to placebo (33), and a phase 3 study for the treatment of liver fibrosis in adults with NASH is currently recruiting (NCT03028740). We have shown here that CVC efficiently blocked the infiltration of Ly6C^hi^ monocytes to the liver in a model of autoimmune cholangitis; however, CVC inhibits also CCR5 and potentially can affect other CCR5-expressing leukocytes. We have found no effect on the hepatic recruitment of T cells, and actually the proportion of hepatic T cells out of all hepatic leukocytes increased in CVC-treated mice without affecting the ratio between hepatic CD4^+^ and CD8^+^ T cells (Figure S2 in Supplementary Material). This increase in T cell proportion is most probably due to the significant effect of CVC on monocyte and monocyte-derived cells infiltration. Of note, two recently published studies showed no effect of CVC on the hepatic recruitment of other CCR5-expressing lymphocyte populations in experimental model of acute liver injury and NASH (31, 34). To exclude that CVC has additional effects on monocyte function, we have exposed LPS-activated THP-1 monocytic cells to various concentration of CVC and found no effect on TNFα secretion (Figure S3 in Supplementary Material), corroborating previous results that demonstrated no effect of CVC on bone marrow-derived macrophage polarization (34).

Primary biliary cholangitis is a classic autoimmune disease characterized by the existence of antigen-specific antibodies as well as antigen-specific T cells with an identified self-antigen expressed specifically by the target tissue—the biliary epithelium (1). Genome-wide association studies suggest a role for innate immunity and in particularly for mononuclear phagocytes in this pathology as manifested by polymorphism in key genes known to be expressed specifically by myeloid cells and having the potential to generate or augment immune response, e.g., *Il12a, Irf5, cd80, Tnfrsf1a*, and *Tnfaip2* (35). We have previously observed an increase in the frequency and absolute numbers of circulating monocytes in patients with PBC compared with sex-matched controls of similar age (20). More important, we have demonstrated that CD14^pos^ monocytes, the human parallel to the mice Ly6C^hi^ monocytes, isolated from patients with PBC are more sensitive to signaling *via* selected toll-like receptors stimulation, resulting in secretion of pro-inflammatory cytokines (20). All of the above suggested an important role for monocyte or monocyte-derived cells in PBC development; however, evidence so far was correlative. Here, we have demonstrated for the first time in the *in vivo* setting the crucial role of Ly6C^hi^ monocytes in the development of this pathology, and by inhibiting the hepatic recruitment of these cells ameliorated all aspects of the disease.

Ursodeoxycholic acid (UDCA) has been the mainstay of therapy for PBC for more than two decades. However, about one-third of patients has persistent elevation of alkaline phosphatase or bilirubin and considered as inadequate responders (36). Obeticholic acid, a farnesoid X receptor agonist, was recently conditionally approved as an add-on therapy in patients with PBC who are inadequate responders to UDCA or who cannot tolerate this drug. However, about 50% of patients with PBC still lack an adequate response to a combination of UDCA and obeticholic acid (37), and therefore, there is still a substantial medical need to develop new therapies for PBC.

In conclusion, Ly6C^hi^ monocyte and their progeny critically contribute to the pathogenesis of PBC and inhibition of their recruitment constitutes an attractive therapeutic avenue in patients non-responding to current available medications. These results reinforce investigation of this therapeutic option in clinical trials.

## Ethics Statement

This study was carried out in accordance with the recommendations of National Institutes of Health policy on the care and use of laboratory animals and the protocol was approved by the Tel-Aviv Sourasky Medical Center Animal Use and Care Committee.

## Author Contributions

EZ and MG designed and guided the research. DR and YG performed the animal experiments and analyzed most of the experiments. PL, YL, IM, AK, PV, EL, CV, ZH, and OS contributed to research design and/or conducted experiments. EB performed the histological disease severity assessment. EZ and DR wrote the manuscript. All the authors reviewed and approved the manuscript.

## Conflict of Interest Statement

EL and PV are employee of Allergan (South San Francisco, CA, USA). The CCR2/CCR5 inhibitor cenicriviroc was kindly provided by Tobira Therapeutics, a subsidiary of Allergan plc. All other authors disclose no conflict of interest related to this study. The reviewer JR and handling Editor declared their shared affiliation.

## Supplementary Material

Figure S1 C-C motif chemokine receptor 2 (CCR2) deficiency or CVC treatment have no influence on the levels of anti-mitochondrial antibody (AMA) titers in the 2-octynoic acid conjugated to bovine serum albumin (2OA-BSA) autoimmune cholangitis model. AMA titers were measured: **(A)** CCR2-deficient mice. **(B)** CVC treatment, 8 weeks following 2OA-BSA immunization by enzyme-linked immunosorbent assay. Results are mean ± SEM (*n* ≥ 10) for each group. **p* < 0.05 and ***p* < 0.01 (unpaired Student’s *t*-test).

Figure S2 The effect of CVC treatment on T cell infiltration into the liver. **(A)** Representative flow cytometry analysis of the percentage T cells out of CD45^+^ cells 8 weeks following immunization with 2-octynoic acid conjugated to bovine serum albumin (2OA-BSA), treated with CVC or vehicle only. **(B)** Graphical summary of flow cytometry analysis shown in panel **(A)**. **(C)** Flow cytometry analysis of CD4/CD8 T cell ratio following 2OA-BSA immunization treated with CVC or vehicle only. **(D)** Graphical summary of flow cytometry analysis shown in panel **(C)**. Results are mean ± SEM (*n* ≥ 10) for each group. **p* < 0.05 (unpaired Student’s *t*-test).

Figure S3 The effect of CVC on macrophage function. THP-1 cells were incubated for 24 h with various concentrations of CVC prior LPS stimulation for 3 h. TNFα concentrations in the soup were measured by enzyme-linked immunosorbent assay.

## Abbreviations

2OA-BSA, 2-octynoic acid conjugated to bovine serum albumin; AMAs, anti-mitochondrial antibodies; CCL2, chemokine (C–C motif) ligand 2; CCR2, C–C motif chemokine receptor 2; Coll1, collagen 1; CVC, cenicriviroc (CCR2/CCR5 inhibitor); CX3CL1, C-X3-C motif chemokine ligand 1 (fractalkine); CX3CR1, C-X3-C motif chemokine receptor 1; DCs, dendritic cells; H&E, hematoxylin and eosin; i.p., intraperitoneal; IFN-γ, interferon gamma; IL-1β, interleukin 1 beta; MoMFs, monocyte-derived macrophages; NASH, non-alcoholic steatohepatitis; PBC, primary biliary cholangitis; PDC-E2, pyruvate dehydrogenase complex-E2; αSMA, alpha smooth muscle actin; TGFβ, transforming growth factor beta; TIMP-1, tissue inhibitor of metalloproteinase 1; TNF-α, tumor necrosis factor alpha; UDCA, ursodeoxycholic acid.
