# Supplementary figures and images for: The Critical Role of Chemokine (C–C Motif) Receptor 2-Positive Monocytes in Autoimmune Cholangitis
Source: Front Immunol. 2018 Aug 15;9:1852. doi: 10.3389/fimmu.2018.01852 (PMC6104446; doi:10.3389/fimmu.2018.01852)

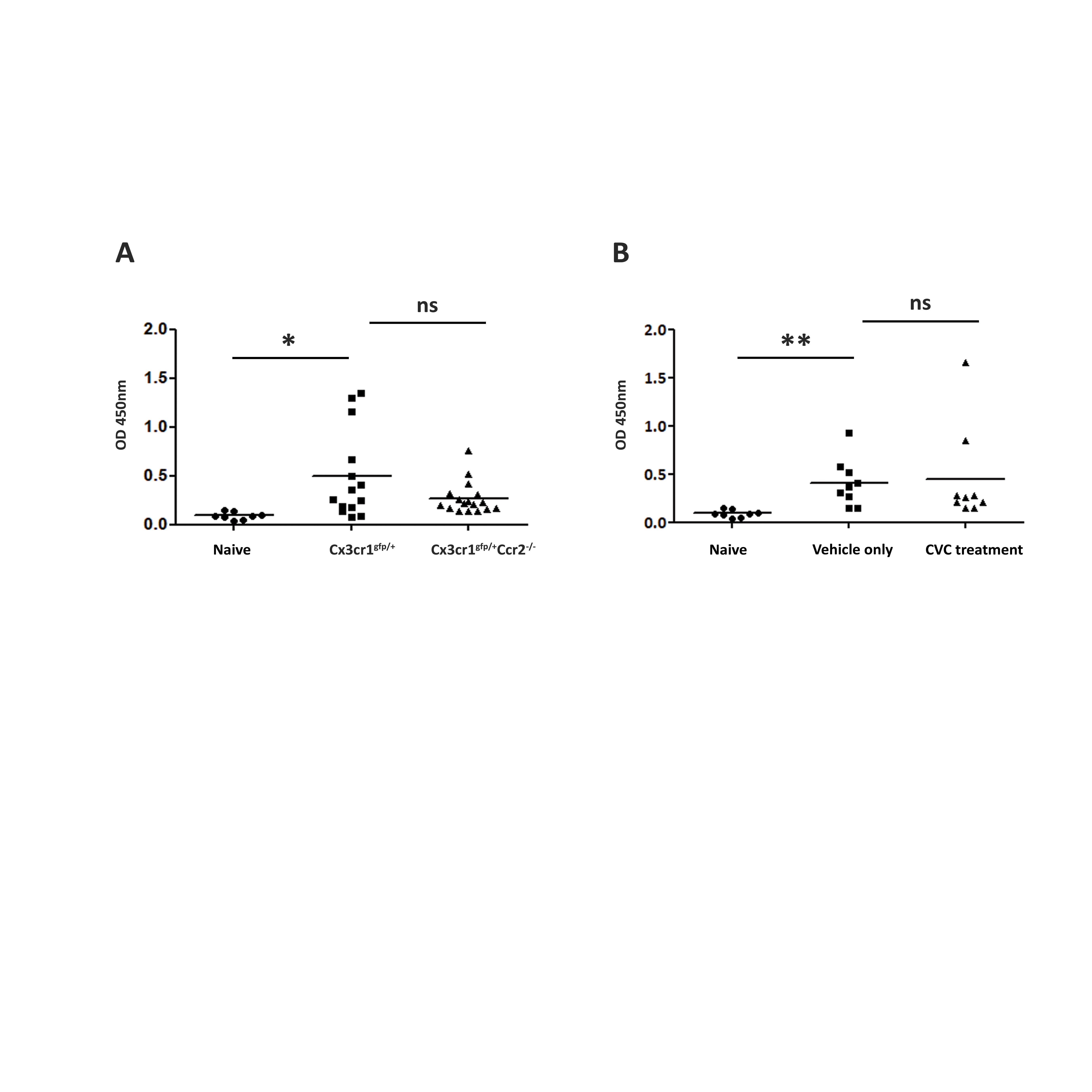

Supplement: Figure S1 — C-C motif chemokine receptor 2 (CCR2) deficiency or CVC treatment have no influence on the levels of anti-mitochondrial antibody (AMA) titers in the 2-octynoic acid conjugated to bovine serum albumin (2OA-BSA) autoimmune cholangitis model. AMA titers were measured: (A) CCR2-deficient mice. (B) CVC treatment, 8 weeks following 2OA-BSA immunization by enzyme-linked immunosorbent assay. Results are mean ± SEM (n ≥ 10) for each group. *p < 0.05 and **p < 0.01 (unpaired Student’s t-test). [file image_1.jpeg]

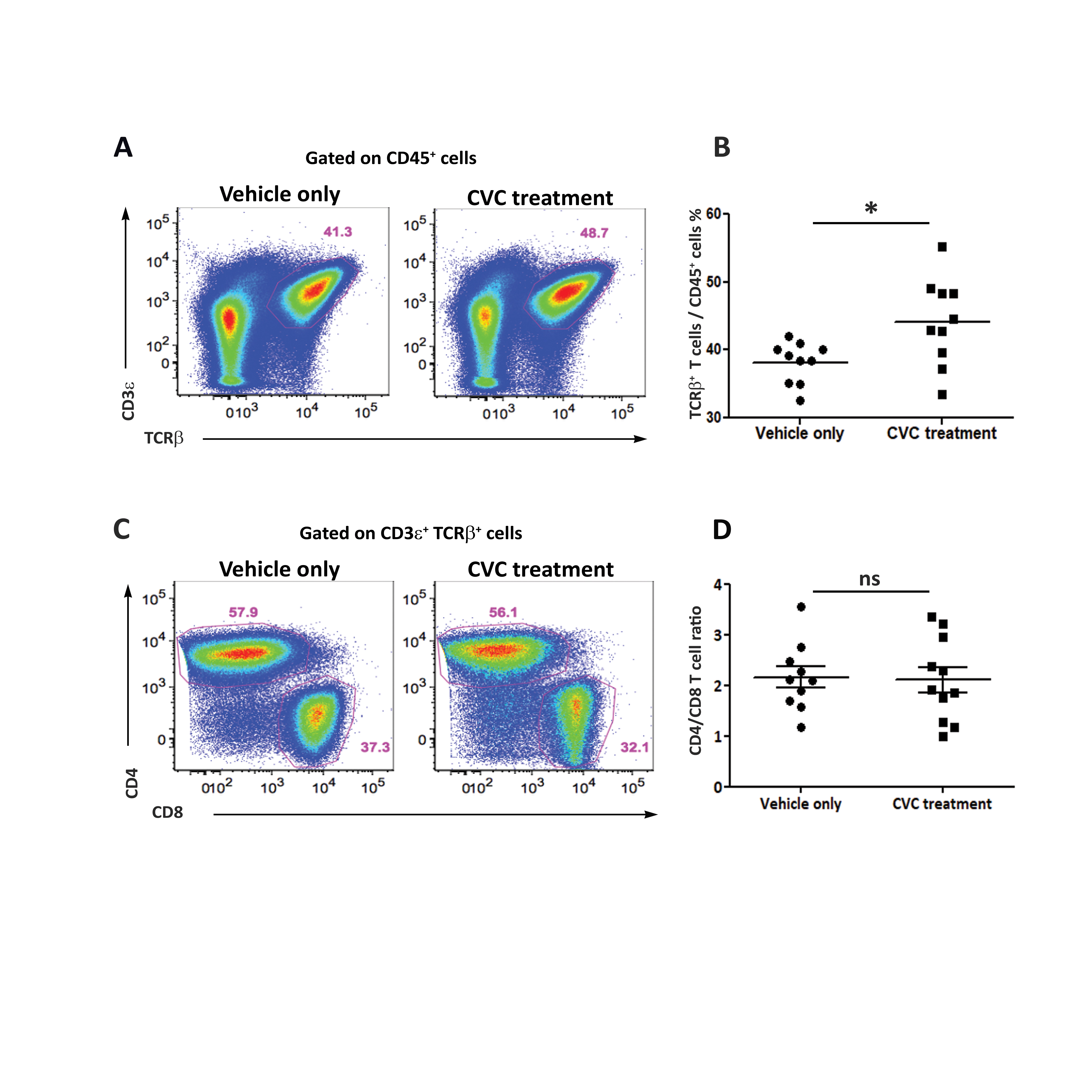

Supplement: Figure S2 — The effect of CVC treatment on T cell infiltration into the liver. (A) Representative flow cytometry analysis of the percentage T cells out of CD45+ cells 8 weeks following immunization with 2-octynoic acid conjugated to bovine serum albumin (2OA-BSA), treated with CVC or vehicle only. (B) Graphical summary of flow cytometry analysis shown in panel (A). (C) Flow cytometry analysis of CD4/CD8 T cell ratio following 2OA-BSA immunization treated with CVC or vehicle only. (D) Graphical summary of flow cytometry analysis shown in panel (C). Results are mean ± SEM (n ≥ 10) for each group. *p < 0.05 (unpaired Student’s t-test). [file image_2.jpeg]

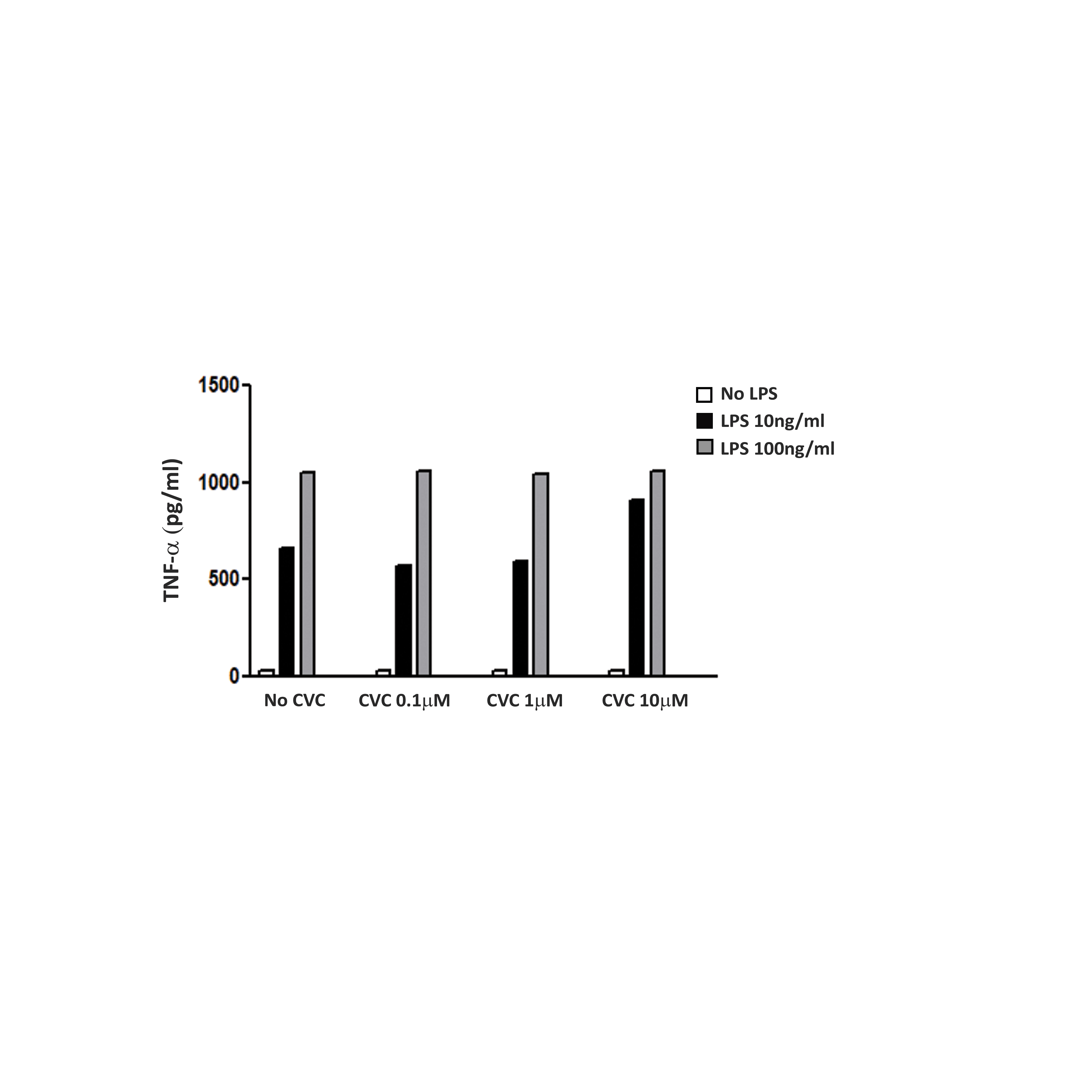

Supplement: Figure S3 — The effect of CVC on macrophage function. THP-1 cells were incubated for 24 h with various concentrations of CVC prior LPS stimulation for 3 h. TNFα concentrations in the soup were measured by enzyme-linked immunosorbent assay. [file image_3.jpeg]
